# Anorexia nervosa as a disorder of the subcortical–cortical interoceptive-self

**DOI:** 10.1007/s40519-022-01510-7

**Published:** 2022-11-10

**Authors:** Lorenzo Lucherini Angeletti, Matteo Innocenti, Federica Felciai, Emanuele Ruggeri, Emanuele Cassioli, Eleonora Rossi, Francesco Rotella, Giovanni Castellini, Giovanni Stanghellini, Valdo Ricca, Georg Northoff

**Affiliations:** 1 Department of Health Sciences, Psychiatry Unit, University of Florence, Largo G. Alessandro Brambilla 3, 50134 Florence, Italy; 2 AOU Careggi Hospital, Psychiatry Unit, Florence, Italy; 3 Department of Health Sciences, Psychology Unit, University of Florence, Florence, Italy; 4 Mental Health Centre, Zhejiang University School of Medicine, Hangzhou, China; 5 Centre for Cognition and Brain Disorders, Hangzhou Normal University, Hangzhou, China; 6 The Royal’s Institute of Mental Health Research & University of Ottawa, Ottawa, ON Canada; 7 Centre for Neural Dynamics, Faculty of Medicine, Brain and Mind Research Institute, University of Ottawa, Ottawa, ON Canada

**Keywords:** Anorexia nervosa, Interoception, fMRI, Self-objectification, Resting-state functional connectivity, Task-induced activity

## Abstract

**Purpose:**

Anorexia nervosa (AN) is characterized by a diminished capacity in perceiving the physiological correlates of interoceptive sensations, namely bodily self-consciousness. Given the neural division of self-processing into interoceptive-, exteroceptive- and mental-self, we hypothesize neural deficits in the interoceptive-processing regions in AN.

**Methods:**

To prove this, we reviewed resting state (rs), task and rest-task studies in AN literature.

**Results:**

Neuronal data demonstrate the following in AN: (i) decreased rs-functional connectivity (rsFC) of subcortical–cortical midline structures (SCMS); (ii) reduced rsFC between medial (default-mode network/DMN and salience network/SN) and lateral (executive-control network/ECN) cortical regions; (iii) decreased rsFC in mainly the regions of the interoceptive-self; (iv) altered activity with overall increased activity in response to sensory/body image stimuli, especially in the regions of the interoceptive-self; (v) lack of a clear task-related distinction between own’s and others’ body image.

**Conclusion:**

These data may indicate that rs-hypoconnectivity between SCMS, as neural correlate of a reduced intero-exteroceptive integration resulting in self-objectification, might be linked to overall increased activity in interoceptive regions during sensory/body image stimuli in AN, engendering an “anxious bodily self.”

**Level of evidence:**

I: Systematic review.

## Introduction

Anorexia nervosa (AN) is a psychopathological disorder closely related to one’s body and its associated somatic, emotional and cognitive features [1]. It is characterized by low body weight, fear of weight gain and distorted perception of own body or persistent lack of recognition of the seriousness of the current low body weight [2], resulting in the highest mortality rate of any psychiatric illness [3]. Patients with AN also report profound disconnection from their own body sensations and emotions [4–6], with consequent difficulty in understanding their own and others’ internal experiences [7, 8].

Indeed, emergent evidence suggests that abnormalities in the feeling of bodily sensations, namely interoception, may play a role in the pathogenesis of this condition, underlining the importance that is attributed to the body in these patients [9, 10]. Interoception refers to the sense of the physiological condition of the body originating from within its internal organs, and body image, namely the perception, feelings, and attitudes one has about one’s body [11–15]. Several studies have identified an altered interoception in its objective and subjective measurements in patients with AN, such as decreases in objective accuracy in detecting internal bodily sensations, i.e., interoceptive accuracy, and in metacognitive awareness of interoceptive accuracy, i.e., interoceptive awareness [16–19].

Recently, neuroscientific research has targeted bodily aspects of self-consciousness, based on the processing of multisensory bodily signals, namely bodily self-consciousness (BSC) [20, 21], and evidence about self-consciousness has also highlighted the importance of interoceptive signals for the self [11, 14, 22, 23]. Nevertheless, most of the studies focused on the manipulation of sources of information coming from outside the body, namely exteroception (i.e., sight, hearing, smell, taste and touch). However, evidence has been put forward that the brain’s perception of internal bodily states, i.e., interoception [13], is equally or even more important for the self [22, 24]. Together, the interoceptive and exteroceptive systems form a common network for BSC [25–27], which might be altered in AN. The difficulty in integrating interoceptive and exteroceptive stimuli from own’s body and the environment may thus underlie a deficit in self-consciousness [28, 29].

One may hence presume that in patients with AN, the experience of their own body is not integrated into their self [30]. Accordingly, AN patients appear to maintain an attitude of “objectification” toward their body, as if their body does not pertain to their self, i.e., not self-related [31, 32]. In this regard, phenomenological research supports the concept that at the origin of the abnormal bodily experience in persons with AN, there is a disorder of the optical-coenaesthetic proportion [31, 32]. In this light, the specific interoceptive alteration detected in patients with AN can be seen as the perception of one’s own body experienced first and foremost as an object being looked at by another, rather than coenaesthetically (interoceptively) apprehended from a first-person perspective. The focus of the optical-coenaesthetic disproportion hypothesis is on data pointing to dampened multisensory integration of interoceptive and exteroceptive signals, highlighting a predominance of the visual afferents toward signals arising within the body [5, 31–35]. However, the exact neural correlates of such body-objectification with body-self disruption are not known yet.

The self can be considered as a complex integration of physical, emotional and cognitive representation of one’s subjective experience engendering one’s identity [28, 29]. Thus, the self plays a pivotal role in the integration of multisensory bodily signals, motivational, emotional, and cognitive aspects. In order to reach a unitary framework for the variety of self-concepts, a recent meta-analysis provided neural evidence for a three-level-self model, which highlights a gradient pattern of self-processing from the internal body to the external environment; this includes interoceptive-, exteroceptive- and mental-self-processing [36].

The pre-requisite for self-processing lays in the integration of sensory signals arising from major survival-based physiological functions (e.g., cardiovascular, respiratory, hunger signals), namely interoceptive-processing. The core hub of interoceptive-processing is in the insula, but it also receives information from subcortical areas like the thalamus. The exteroceptive-processing serves to link our internal body to the external non-bodily stimuli; it also has its foundations in the insula, with the participation of the anterior medial prefrontal cortex (AMPFC), superior parietal lobule (SPL) and premotor cortex (PMC). Finally, to introduce self-relatedness to external non-bodily stimuli, mental-self-processing recruits more extensive brain regions such as the medial prefrontal cortex (MPFC) and posterior cingulate cortex (PCC), in addition to insula, AMPFC and SPL [36]. Since this model attributes a pivotal role to the interoceptive-processing, indicating that integrated internal sensory signals could be the key underlying self-processing, and given that interoceptive-processing and awareness are deficient in AN, the main hypothesis of the present study is that the lowest layer of self, the interoceptive self and its neural correlates should be altered in these patients.

The aim of our study is to review fMRI literature about AN starting with resting-state studies, up to studies that analyzed brain activity under task conditions. Indeed, several fMRI studies have highlighted altered activity in various regions belonging to the default mode network (DMN), salience network (SN) and executive control network (ECN) during both rest and task states [37, 38]. As it is still unclear how the neural changes reported both at rest and in task conditions are related to the changes in especially the interoceptive self in AN, one specific aim of our study is to extrapolate the neural activity of the regions directly involved in self-processing—according to the three layers topography of self—in order to better understand the link between self and body in AN [36]. To do this, we first analyze the whole brain resting-state activity, i.e., resting-state functional connectivity (rsFC), in patients with AN with respect to controls. Secondly, we will apply a specific focus to the resting activity of the regions involved in self-processing. Finally, we analyze the activity of the same regions when stimulated by AN-specific tasks.

## Materials and methods

### Eligibility criteria

The study selection followed a three-stage approach in order to search: (1) studies using resting-state fMRI in AN; (2) task-evoked fMRI studies in AN; (3) combined rest and task fMRI studies in AN. Inclusion criteria were as follows: (i) fMRI studies on (ii) patients with ongoing AN (acute-phase AN), compared to (iii) a control group. Exclusion criteria were as follows: (i) other neuroimaging studies (e.g., PET, dMRI, etc.); (ii) the presence of patients in remission or recovered from AN; (iii) patients with subclinical or atypical AN; and iv) the lack of a control group, i.e., healthy controls (HC) with no current presence or history of eating disorders.

### Search strategy

The systematic literature search and study selection approach followed PRISMA guidelines. To be included in this work, studies had to be published in peer-reviewed scientific journals in order to be considered of comparable quality. PubMed and Web of Science were the source of information and an online search was conducted using the following strings of research for the time frame up to September 2022: (“anorexia nervosa” OR “eating disorder”) AND (“insula” OR “interoception”) AND “fMRI” AND “resting state”; (“anorexia nervosa” OR “eating disorder”) AND (“insula” OR “interoception”) AND “fMRI” AND (“task-evoked activity” OR “task/stimulus-induced”). Filters to exclude reviews and meta-analysis were added. To complete the search strategy, once the eligible studies were identified, we reviewed the references of these studies in order to find other studies eligible for review. Finally, we went over reviews and meta-analyses already in the literature to find additional studies that we could have been left out [37, 38].

### Study selection

Three authors (LLA, MI and FF), working independently, selected the articles at each stage of the review (identification, screening and inclusion) by using Cochrane’s online software for systematic reviews, Covidence (Covidence systematic review software, n.d.). Authors resolved disagreements through discussion and consensus, and any remaining disagreements were resolved by another author (GN).

**Fig. 1:**
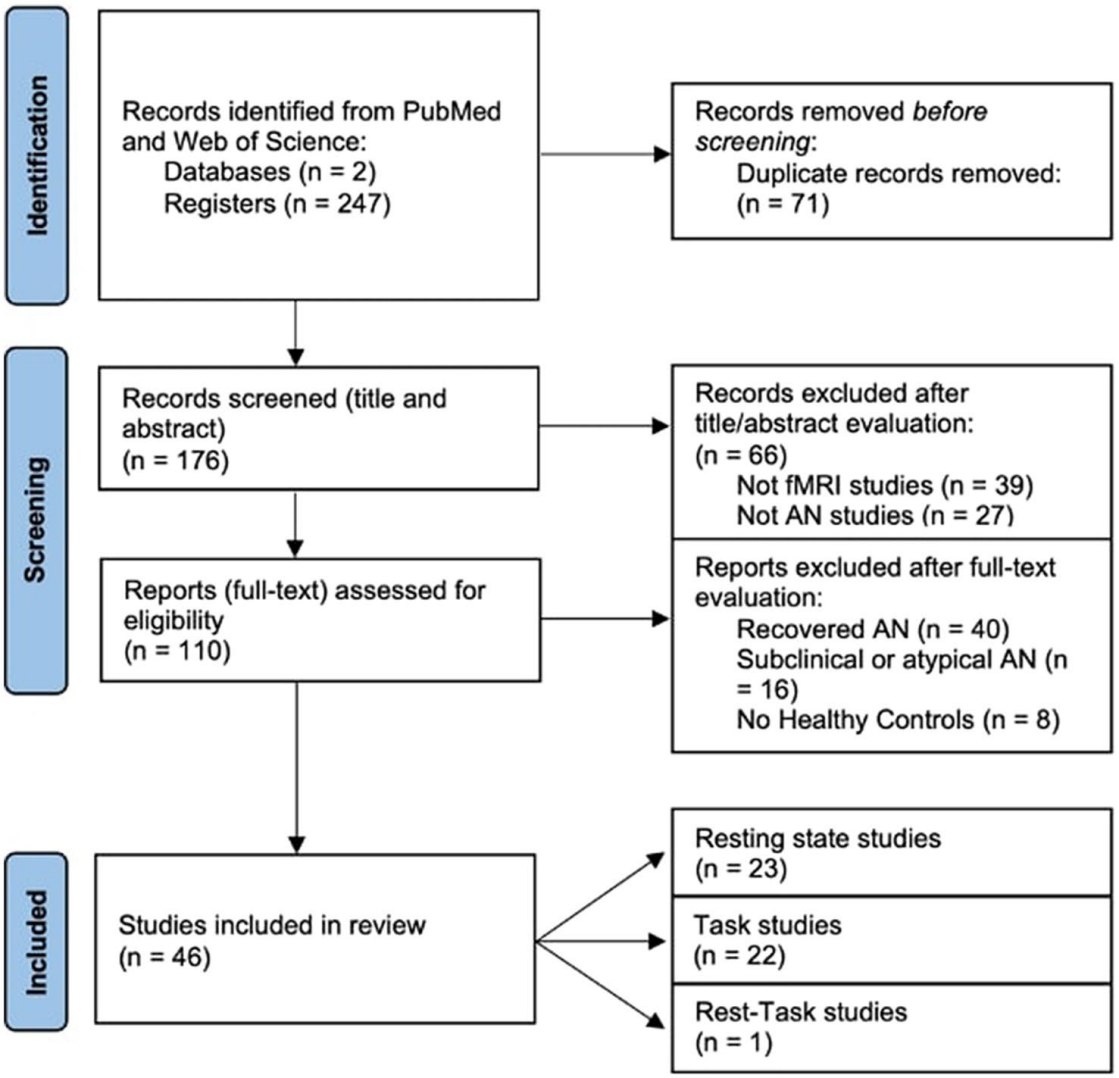
PRISMA flowchart of the article selection process

The process of selection of the studies is shown in Fig. 1. Database searches allowed to identify 247 articles. The articles were exported to Mendeley to eliminate duplicates (*n* = 71). We therefore screened titles and abstracts of 176 studies, from which we excluded 66 articles that did not use fMRI (*n* = 39) or that did not include a sample study with AN (*n* = 27). This led us to the identification of 110 articles, whose eligibility has been assessed by reading the full text. The main reason for exclusion at the phase of “full text reading” was the presence of analyzed samples not corresponding to patients with acute stage AN, such as recovered AN (*n* = 40) or subclinical AN (*n* = 16) or the absence of HC (*n* = 8). After the full texts were screened, 46 articles were found to meet the inclusion criteria subdivided as follows: (a) twenty-three resting-state studies, (b) twenty-two task-related studies and (c) one rest-task study (see details in Fig. 1).

Since AN is a disorder with an early onset, i.e., around the age of 13, it has been studied in patients since adolescence [1]. In particular, adolescent AN studies were found to be higher among the resting-state studies, probably due to the different and less complicated methodological approach required by the resting-state studies compared to the task studies. Therefore, given the presence in the literature of a differentiation in the AN samples between patients with a mean age under or over 18 years old, we decided to analyze the extrapolated resting-state studies dividing them according to age.

### Data extraction and characteristics of the studies

Following the PRISMA guidelines, data were extracted in tables independently by three authors (LLA, MI and ER). The methodological quality of the studies was assessed by these authors, working first separately and then together. From the selected studies, we extrapolated the: (a) type of AN (restricting or binge-purging type), (b) total sample (AN vs HC), (c) mean age of AN group (summarizing whether they were with age above or below 18 years old), (d) mean BMI of AN group, (e) duration of illness (reported in months or years depending on the study) and f) type of paradigm (see Table 1). It is important to consider that the diversity in the methodologies within the studies included in this systematic review precluded the completion of a meta-analysis.

**Table 1:** Studies included in the review

	RS Studies	Type of AN	Total sample	Age	Mean BMI in AN	Illness duration	Type of paradigm
Rest adolescence AN							
1	Boehm et al. [52]	33 Res-AN 2 BP-AN	35 AN 35 HC	< 18	14.7 ± 1.2	18.9 ± 27.0 (in months)	Resting state (RS)
2	Ehrlich et al. [39]	33 Res-AN 2 BP-AN	35 AN 35 HC	< 18	14.7 ± 1.2	18.9 ± 27.0 (in months)	RS
3	Gaudio et al. [51]	Res-AN (early stages)	16 AN 16 HC	< 18	16.2 ± 1.2	4.0 ± 1.8 (in months)	RS
4	Geisler et al. [39]	33 Res-AN 2 BP-AN	35 AN 35 HC	< 18	14.7 ± 1.2	18.9 ± 27.0 (in months)	RS
5	Lord et al., 2016	33 Res-AN 2 BP-AN	35 AN 35 HC	< 18	14.7 ± 1.2	18.9 ± 27.0 (in months)	RS
6	Gaudio et al. [42]	Res-AN (early stages)	15 AN 15 HC	< 18	16.1 ± 1.2	4.0 ± 1.8 (in months)	RS
7	Lotter et al. [50]	21 Res-AN 1 BP-AN	22 AN 22 HC	< 18	15.7 ± 1.5	1.6 ± 1.3 (in years)	RS
8	Myrvang et al. [57]	Res-AN	29 AN 27 HC	< 18	16.3 ± 1.7	–	RS
9	Via et al. [43]	22 Res-AN 8 BP-AN	30 AN 17 HC	< 18	16.9 ± 1.0	–	RS
Rest adult AN							
10	Favaro et al.[58]	–	29 AN 26 HC	> 18	14.5 ± 2.3	74.5 ± 82.4 (in months)	RS
11	Amianto et al. [44]	Res-AN	12 AN 10 HC	> 18	16.2 ± 0.9	11.0 ± 5.0 (in months)	RS
12	Kullmann et al. [59]	–	12 AN 14 HC	> 18	15.5 ± 1.5	–	RS
13	Lee et al. [56]	6 Res-AN 12 BP-AN	18 AN 20 HC	> 18	16.0 ± 1.7	3.8 ± 2.6 (in years)	RS
14	Phillipou et al. [53]	-	26 AN 27 HC	> 18	16.6 ± 1.2	6.4 ± 7.4 (in years)	RS
15	Biezonski et al. [45]	7 Res-AN 8 BP-AN	15 AN 16 HC	> 18	17.1 ± 0.2	53.8 ± 7.1 (in months)	RS
16	Scaife et al., 2016	Res-AN	12 AN 16 HC	> 18	15.4 ± 1.9	10.3 ± 5.2 (in years)	RS
17	Cha et al. [47]	–	22 AN 18 HC	> 18	17.3 ± 1.2	–	RS
18	Canna et al. [60]	–	15 AN 16 HC	> 18	16.8 ± 1.6	7.9 ± 6.9 (in years)	RS
19	Via et al. [55]	Res-AN	20 AN 20 HC	> 18	16.9 ± 1.2	85.2 ± 76.8 (in months)	RS
20	Uniacke et al., 2018	14 Res-AN 11 BP-AN	25 AN 25 HC	> 18	16.5 ± 2.0	3.5 ± 2.8 (in years)	RS
21	Spalatro et al. [61]	–	25 AN 17 HC	> 18	15.9 ± 0.9	–	RS
22	Haynos et al. [48]	Res-AN	19 AN 19 HC	> 18	16.9 ± 1.3	8.0 ± 3.7 (in years)	RS
23	De la Cruz et al. [49]	–	22 AN 26 HC	> 18	15.1 ± 1.4	–	RS
Intero-exteroceptive tasks							
1	Kerr et al. [62]	Res-AN	15 AN 15 HC	> 18	19.8 ± 0.8	–	Interoceptive attention task (IAT): heart, stomach, bladder
2	Kerr et al. [63]	Res-AN	20 AN 20 HC	< 18	19.8 ± 0.8	–	IAT: heart, stomach, bladder
3	Vocks et al. [67]	Res-AN	12 AN 12 HC	> 18	14.0 ± 1.7	84.5 ± 42.8 (in months)	Gustatory stimulation (GS)
4	Frank et al. [73]	Res-AN	21 AN 23 HC	> 18	16.1 ± 1.0	6.4 ± 5.2 (in years)	GS
5	McFadden et al. [70]	Res-AN	20 AN 24 HC	> 18	16.0 ± 1.0	6.7 ± 6.2 (in years)	GS
6	Frank et al. [74]	Res-AN	21 AN 27 HC	> 18	16.0 ± 1.1	–	GS
7	Monteleone et al. [66]	16 Res-AN 4 BP-AN	20 AN 20 HC	> 18	17.3 ± 1.0	7.6 ± 6.7 (in years)	GS
8	Frank et al. [75]	Res-AN	56 AN 52 HC	< 18	15.8 ± 0.8	–	GS
9	Olsavsky et al. [69]	Res-AN	28 AN 43 HC	> 18	16.1 ± 1.0	–	GS
10	Jiang et al. [68]	Res-AN	14 AN 12 HC	> 18	15.8 ± 0.9	–	Olfactory stimulation (OS)
11	Bar et al., 2013	Res-AN	19 AN 19 HC	> 18	16.9 ± 1.2	7.5 ± 3.3 (in years)	Thermal pain stimulation (TPS)
12	Bar et al., 2015	Res-AN	15 AN 15 HC	> 18	17.2 ± 1.2	22.5 ± 14.1 (in months)	TPS
13	Davidovic et al. [71]	21 Res-AN 4 BP-AN	25 AN 25 HC	> 18	16.3 ± 1.0	4.1 ± 3.5 (in years)	Tactile stimulation (TS)
Body image tasks							
14	Seeger et al. [79]	–	3 AN 3 HC	< 18	15.3 ± 0.6	–	Self-normal/distorted body image task (S–N/D BIT)
15	Wagner et al. [80]	Res-AN	13 AN 11 HC	< 18	14.6 ± 1.3	–	S–N/D BIT
16	Sachdev et al. [77]	5 Res-AN 5 BP-AN	10 AN 10 HC	> 18	16.4 ± 1.0	–	Self-other body image task (S–O BIT)
17	Friederich et al. [83]	10 Res-AN 7 BP-AN	17 AN 18 HC	> 18	15.6 ± 1.4	7.2 ± 4.3 (in years)	S–O BIT
18	Vocks et al.[67]	7 Res-AN 6 BP-AN	13 AN 27 HC	> 18	15.7 ± 1.2	7.2 ± 6.2 (in years)	S–O BIT
19	Mohr et al. [84]	–	16 AN 16 HC	> 18	15.9 ± 1.2	–	S–N/D BIT
20	Miyake et al. [81]	11 Res-AN 11 BP-AN	22 AN 11 HC	> 18	15.3 ± 1.8 *(Res-AN)* 15.9 ± 1.9 *(BP-AN)*	4.1 ± 3.3 (Res-AN) 7.9 ± 4.4 (BP-AN) (in years)	S–N/D BIT
21	Castellini et al. [82]	Res-AN	18 AN 19 HC	> 18	16.0 ± 1.4	5.6 ± 6.8 (in years)	S–N/D BIT
22	Via et al. [55]	Res-AN	20 AN 20 HC	> 18	16.9 ± 1.2	85.2 ± 76.8 (in months)	S–O BIT
Rest-task studies							
1	Via et al. [55]	Res-AN	20 AN 20 HC	> 18	16.9 ± 1.2	85.2 ± 76.8 (in months)	RS + S–O BIT

Adolescence = sample mean age < 18 years old; Adult = sample mean age > 18 years old

*AN* Anorexia Nervosa, *HC* Healthy Controls, *Res-AN* Restricting type AN, *BP-AN* Binge Purging type AN

#### rsfMRI studies in AN

For resting state, of the 247 studies initially retrieved, only 23 fulfilled the above-described inclusion criteria. Of these, (i) nine studies with an AN and HC sample aged less than 18 years (adolescent AN), and (ii) 14 studies with an AN and HC sample aged over 18 years (adult AN) (T1).

Furthermore, in order to analyze brain activity following the tripartite model of self-processing [36], we have divided the resting-state activity into: (i) subcortical-to-cortical rsFC, i.e., all the reported rsFCs in which the subcortical structures and their functional connectivity with cortical structures are involved, representing the interoceptive-processing; (ii) cortical-to-cortical rsFC, concerning all rsFCs between cortical structures, which instead characterizes the subsequent levels of self-processing, i.e., exteroceptive- and mental-self-processing.

Finally, we extrapolated the activity of the brain regions responsible for the different layers of self-processing, i.e., interoceptive-, exteroceptive-, and mental-self, starting from the lowest level (interoceptive) up to the propagation of these regions in the following layers (exteroceptive- and mental-self). In this way, we subsequently used these as regions-of-interest (ROI) in the task studies.

#### Task-evoked fMRI studies in AN

In the second stage, we identified 22 task-related studies in AN using a literature search via PubMed and Web of Science as previously illustrated. Afterward, we differentiated these studies in two categories on the basis of stimulus type and induced outcome (T1): (i) 13 studies with interoceptive and exteroceptive stimuli: *intero-exteroceptive tasks*, in order to evaluate the activity during the integration of bodily information coming from the inside and the outside in AN patients; (ii) nine studies with body image stimuli: *body image tasks,* in which AN patients are shown images of (a) their own body as it appears; (b) their own body in thinner conditions; (c) their own body in fatter conditions; and (d) other subjects’ body. The latter paradigm is commonly used in patients with AN, and we decided to consider it because it encompasses the integration of intero-exteroceptive information and the mental idea that patients have of themselves. In these studies, we investigated how the previously defined rs-ROIs involved in the three different layers of self-processing responded in accordance with the different tasks in AN patients with respect to healthy controls. This step allowed us to make hypotheses about the changes in activity from rest to task and how they might be intrinsically related. We could not differentiate on the basis of age in the task condition, as only four out of twenty-two studies had an AN sample under the age of 18 (T1).

#### Combined rest-task studies in AN

In the third stage, we obtained one fMRI study including both rest and task conditions (T1). This was the only study which gave us information about the rest-task relationship in the same AN subjects to test our initial hypothesis.

## Results

### Subcortical-to-cortical functional connectivity in resting state

#### Adolescent AN

**Fig. 2:**
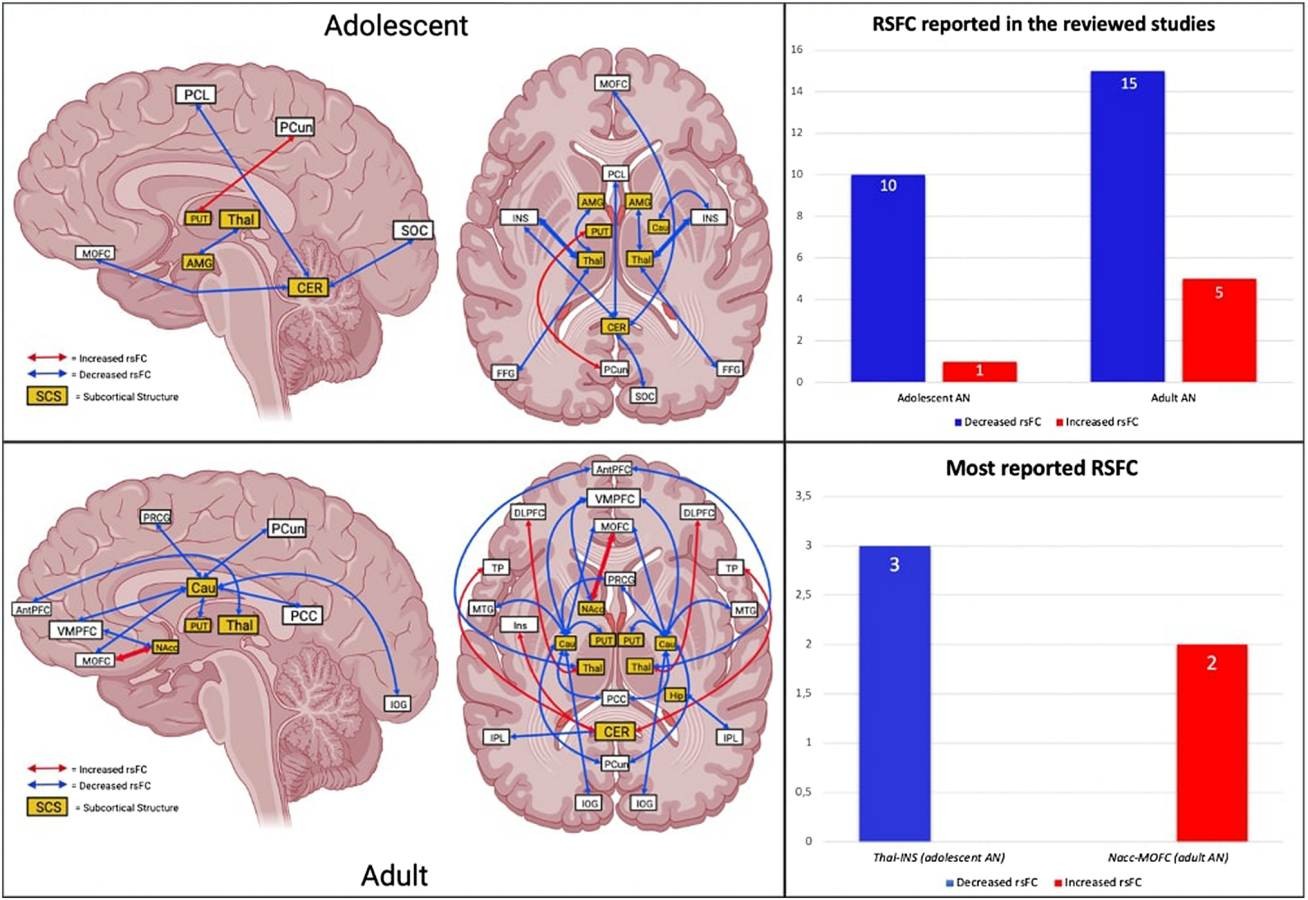
Subcortical-to-cortical resting-state studies, adolescent (upper) and adult (lower) with AN. *MOFC* medial orbitofrontal cortex, *PCL* paracentral lobule, *PCun* precuneus, *SOC* superior occipital cortex, *AMG* amygdala, *PUT* putamen, *Thal* thalamus, *CER* cerebellum, *INS* insula, *FFG* fusiform gyrus, *VMPFC* ventromedial prefrontal cortex, *AntPFC* anterior prefrontal cortex, *PRCG* precentral gyrus, *Cau* caudate, *PCC* posterior cingulate cortex, *IOG* inferior occipital gyrus, *DLPFC* dorsolateral prefrontal cortex, *TP* temporal pole, *MTG* middle temporal gyrus, *NAcc* nucleus accumbens, *IPL* inferior parietal lobule

In the nine reviewed studies about adolescents with AN, five subcortical regions were found to have altered activity at rest with respect to controls, namely thalamus, amygdala (AMG), putamen, caudate and cerebellum. Among them, four regions (i.e., thalamus, AMG, caudate and cerebellum) show decreased rsFC with cortical areas [39–43], whereas putamen is observed to have increased connectivity with PCun [43]. Thalamus is reported to have decreased rsFC with the insula in three different studies [39–41]. Likewise, caudate have also shown reduced rsFC with right insula [43]. Moreover, thalamus is reported to have decreased rsFC with AMG and fusiform gyrus (FFG) [39]. Finally, Gaudio and colleagues (2018) found decreased cerebellum rsFC with anterior (medial orbitofrontal cortex/MOFC, insula and paracentral lobule/PCL) and posterior cortical regions (superior occipital cortex/SOC) [42] (Fig. 2).

#### Adult AN

In adult AN, in addition to four subcortical regions already altered in adolescent AN (i.e., thalamus, putamen, caudate and cerebellum), the reviewed studies also show altered rsFC of the nucleus accumbens and hippocampus with cortical regions [44–49]. Besides, it has been observed an overall decreased rsFC between subcortical and cortical regions, consistent with what reported in adolescent studies. Caudate seems to be a central hub, showing reduced synchronization with anterior (Ventromedial prefrontal cortex/VMPFC, precentral gyrus/PRCG, middle temporal gyrus/MTG) and posterior (posterior cingulate cortex/PCC, precuneus/PCun, inferior occipital gyrus/IOG) cortical regions [48]. Similarly, also thalamus and nucleus accumbens show decreased rsFC with anterior regions, i.e., anterior prefrontal cortex (AntPFC) and VMPFC [45, 48], whereas cerebellum and hippocampus are reported to have diminished connectivity with inferior parietal lobule (IPL) [44, 49]. In these studies, only five increased rsFC are observed, with cerebellum and thalamus showing enhanced rsFC mainly with lateral cortical areas, namely left insula, temporal pole (TP) and dorsolateral prefrontal cortex (DLPFC) [44, 45], while on the contrary nucleus accumbens is being reported as highly connected with MOFC in two different studies [46, 47] (Fig. 2).

### Cortical-to-cortical functional connectivity in resting state

#### Adolescent AN

**Fig. 3:**
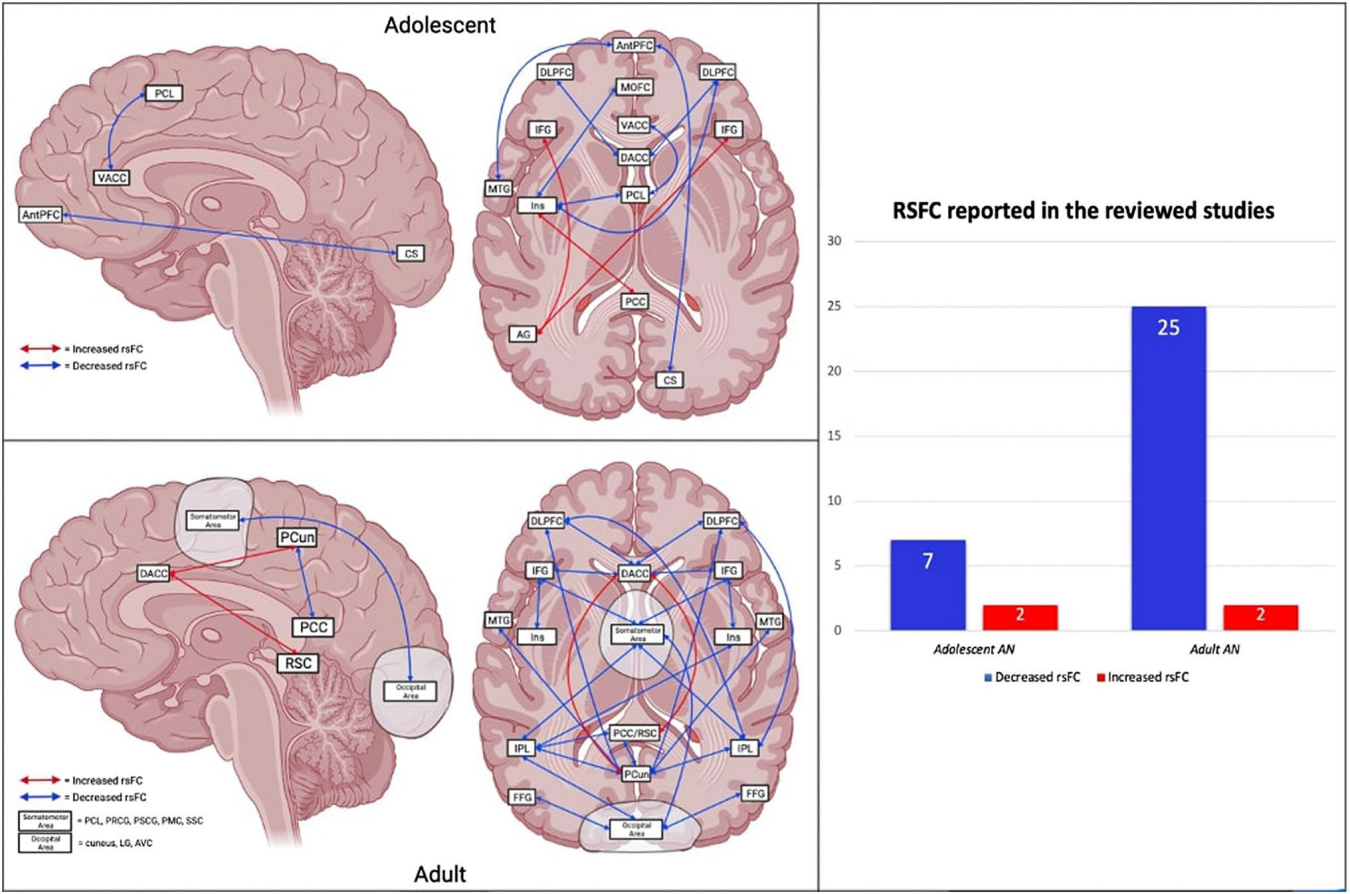
Cortical-to-cortical resting-state studies, adolescent (upper) and adult (lower) with AN. *MOFC* medial orbitofrontal cortex, *VACC* ventral anterior cingulate cortex, *DACC* dorsal anterior cingulate cortex, *PCL* paracentral lobule, *PCun* precuneus, *IFG* inferior frontal gyrus, *INS* insula, *AG* angular gyrus, *CS* calcarine sulcus, *FFG* fusiform gyrus, *AntPFC* anterior prefrontal cortex, *PRCG* precentral gyrus, *PSCG* postcentral gyrus, *PMC* premotor cortex, *SSC* somatosensory cortex, *PCC* posterior cingulate cortex, *RSC* retrosplenial cortex, *DLPFC* dorsolateral prefrontal cortex, *MTG* middle temporal gyrus, *IPL* inferior parietal lobule, *LG* lingual gyrus, *AVC* associative visual cortex

Altered rsFC are reported among cortical regions in adolescent AN. In particular, anterior regions seem to be mainly involved, reporting overall decreased rsFC. More precisely, left insula is observed to have reduced rsFC with MOFC, PCL and right DLPFC [50]. Ventral and dorsal anterior cingulate cortex (VACC, DACC) also show decreased rsFC with PCL and bilateral DLPFC [42, 51], whereas AntPFC has reduced rsFC with left MTG and right calcarine sulcus [50]. Only two increased rsFC are reported in a fMRI study between left angular gyrus (AG) and bilateral inferior frontal gyrus (IFG), and between left insula and PCC [52] (Fig. 3).

#### Adult AN

Various fMRI studies show altered rsFC between cortical regions in adult AN respect to controls. Consistent with adolescent AN studies, overall reduced rsFC is reported. In particular, medial cortical regions show decreased rsFC with lateral cortical regions. According to a recent fMRI study, PCun seems to play a key role, reporting diminished connectivity with bilateral IPL, MTG and DLPFC [49]. Similarly, the somatomotor area (comprehending PCL, PRCG, PoStCentral Gyrus/PSCG, PreMotor Cortex/PMC and SomatoSensory Cortex/SSC) is observed to have reduced FC with bilateral IPL, IFG and the occipital area (including cuneus, Lingual Gyrus/LG, Associative Visual Cortex/AVC) [49, 53, 54]. The latter area also show decreased rsFC with bilateral FFG and left IPL [49, 54]. Moreover, PCC is also reported to have diminished rsFC with left IPL [55]. Notably, insula, despite showing more altered rsFC in both subcortical–cortical/cortical–cortical adolescent AN studies, appears to have few reduced connections with IFG and left IPL [49, 54]. Lee and colleagues (2014) found DACC to be a central hub in patients with AN and also reported reduced rsFC with lateral cortical regions (DLPFC and IFG), whereas they observed two increased rsFC with medial cortical regions, namely retrosplenial cortex (RSC) and PCun [56]. These results appear to be in contrast with other studies. However, it is important to take into account that in this study the 66% of the patients were binge-purging subtype, whereas most of the studies included in the present review enrolled patients with AN restricting subtype (Fig. 3).

### Summary of subcortical-to-cortical and cortical-to-cortical rsfc in adolescent and adult AN

Taking everything into account, resting-state findings show (i) almost total hypoconnectivity at rest in patients with AN compared to HCs; (ii) decreased rsFC in subcortical–cortical regions in patients with AN, with thalamus and caudate as key altered hubs; (iii) overall decreased activity between subcortical–cortical midline structures (SCMS); and (iv) a midline-lateral reduced rsFC in DMN, SN and ECN cortical–cortical regions in patients compared to healthy controls.

### Abnormal resting state activity in the three layers topography of self

As a second step of our investigation, we now focus on the altered neural regions in AN which are directly involved in the three different layers of self-processing following the three-layer topography of self [36]. This approach may lead to a better understanding on the role of the self and its various levels of processing in AN and its etiopathogenesis.

**Fig. 4:**
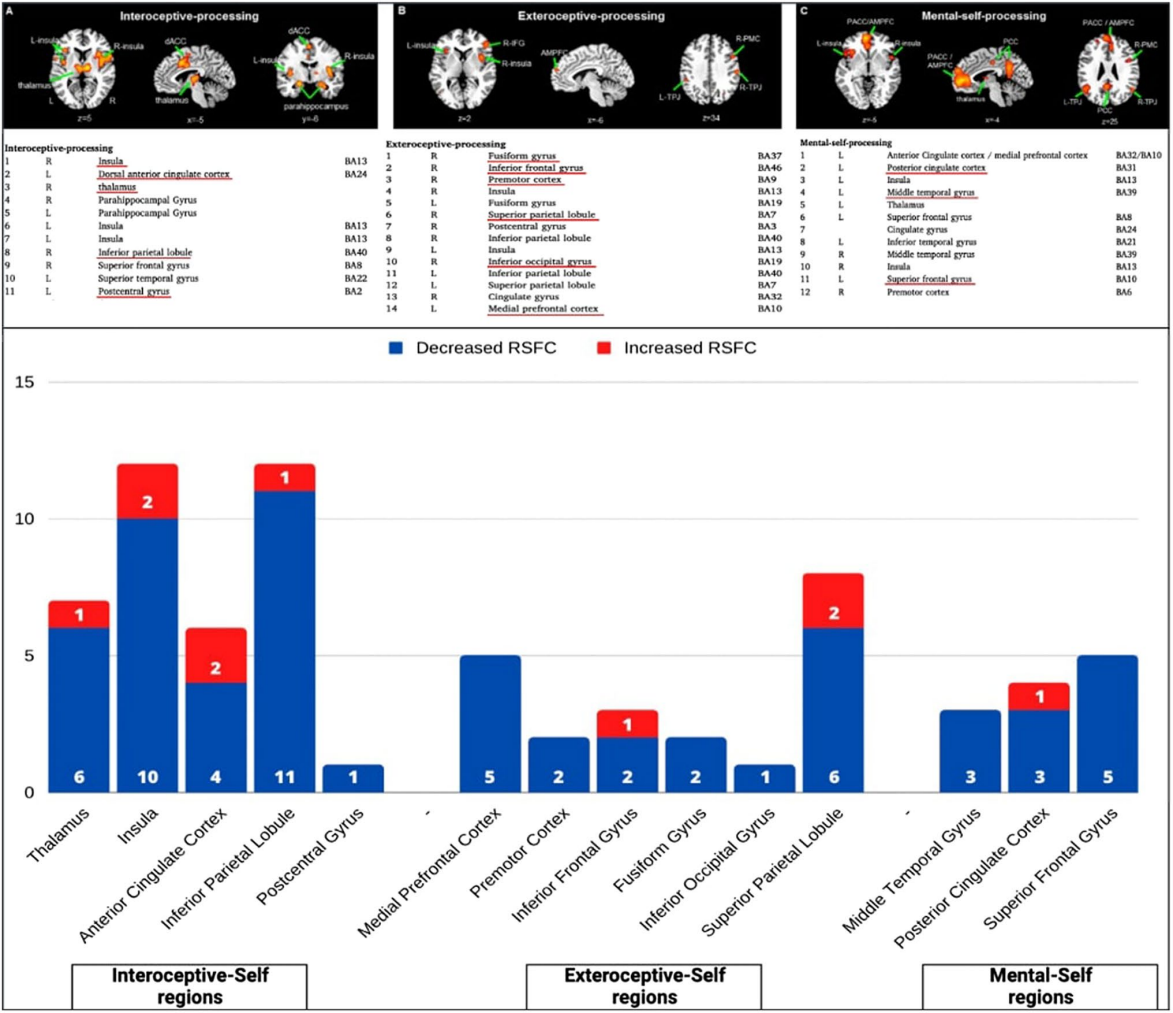
Three-layer topography of self-neural regions: A. Interoceptive-processing, B. Exteroceptive-processing, C. Mental-self-processing (upper); 36 Number of reported abnormal rsFCs in the three-layer topography of self-neural regions in the reviewed studies (lower)

Several rsfMRI studies reported altered rsFC among AN group in the regions involved in the interoceptive-self. More in detail, among the reviewed studies, insula and IPL show twelve altered connections at rest, whereas thalamus, ACC and PSCG display seven, six and one altered rsFC, respectively, with an overall decreased activity reported in the just mentioned regions (Fig. 4).

Going forward, various regions associated with the exteroceptive-processing are reported to have altered activity too. In particular, MPFC and SPL (including parts of the PCun) show altered rsFC (i.e., five and seven respectively) with general reduced rsFC compared to healthy controls. Moreover, IFG (three altered rsFC reported), PMC and FFG (observed to have two altered rsFC) and IOG (one rsFC reported) do show altered connections at rest (Fig. 4).

Finally, the mental-self-processing layer does not seem to report many altered neural regions in patients with AN compared to controls. More precisely, only SFG (which includes parts of the DLPFC), PCC and MTG are reported to have abnormal functional connectivity at rest (mainly decreased rsFC here too), showing five, four and three altered rsFC, respectively, in the reviewed studies (Fig. 4).

As an overall, compared to what is seen in whole-brain resting state activity, most of the altered regions observed at rest in patients with AN are neural regions closely related to the processing of the self. This seems to confirm our initial hypothesis that proposes AN as a disturbance of the self. In particular, we observe the greatest alterations at rest in the regions delegated to the interoceptive-processing, suggesting that AN concerns a lack of integration of information concerning one’s own body in the self. This is supported by the clinical practice as patients often report an objectified vision of their own body, disconnected from their own self [30, 31]. This aspect may be strictly linked with their body misperception (and/or body-self disruption).

### Task-evoked activity in abnormal resting state regions of the three layers topography of self

Finally, in order to understand how the hypoconnectivity observed during the resting state may play a role in brain activity in task conditions, in the third step of our research, we examined the activity of the key brain regions in the three processing layers of the self.

### Intero-exteroceptive tasks—abnormal responsivity to sensory stimuli

There are only two studies in the literature that have investigated interoception in patients with AN. These studies applied an interoceptive attention task, asking patients to focus on sensations coming from the heart, stomach, and bladder [62, 63]. From these studies, the fundamental role of the insula in the processing of interoceptive stimuli is observed. In particular, right insula is reported to have increased activity during heart and stomach attention. Alongside, also MPFC and ACC are observed to be hyperactivated during heart and stomach sensations, respectively [62, 63]. On the contrary, PCun shows decreased activity across all three interoceptive attention domains, with left insula reporting reduced activations when focusing on stomach sensations [62, 63].

In order to evaluate the activity during the integration of bodily information coming from the inside and the outside in patients with AN, we included studies investigating interoception using external stimulation connected to the five exteroceptive senses, i.e., gustatory, thermal, pain, olfactory, tactile stimulation [64]. Several studies also highlight the role of insula in integrating bodily information. More precisely, right insula is reported to have enhanced activity during heat and taste perception tasks and in the presence of a high prediction error [65, 66], whereas left insula shows increased activity only when, during the perception of taste, an elevated prediction error is generated [67]. Otherwise, left insula shows an overall decreased activity, especially during heat perception and olfactory stimulation [65, 68]. The other regions assigned to the interoceptive processing appear to have an overall increased activity. Precisely, right thalamus shows hyperactivity during gustatory perception [66], as well as ACC in the presence of a high prediction error [63, 69]. On the contrary, the latter, together with PSCG, is observed to have diminished activity during the perception of a taste [70].

**Fig. 5:**
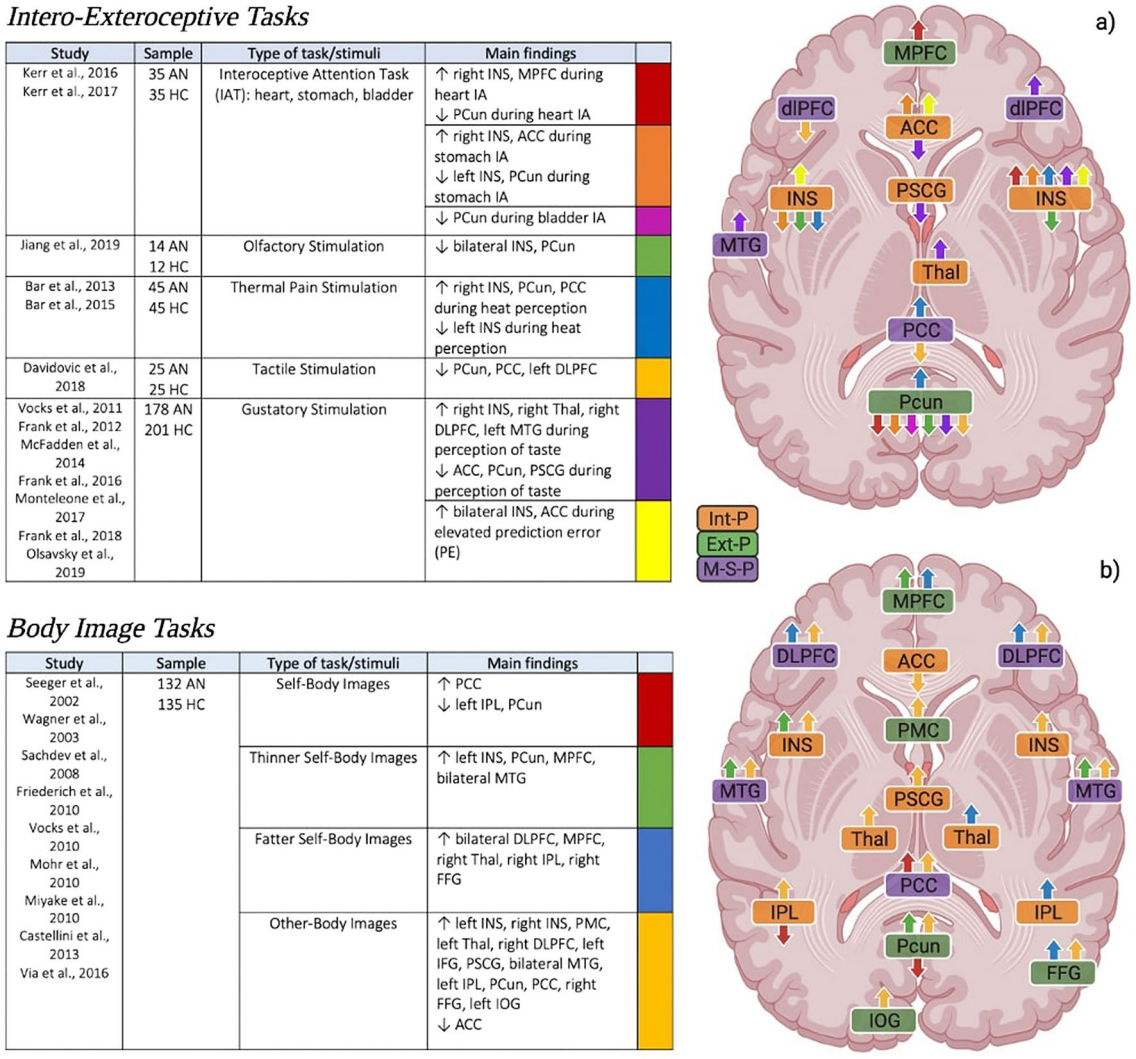
Task-related activity in abnormal resting state regions during **a** intero-exteroceptive and **b** body image tasks. *Int-P* Interoceptive-processing, *Ext-P* Exteroceptive-processing, *M-S-P* Mental-self-processing, *MPFC* medial prefrontal cortex, *ACC* anterior cingulate cortex, *PCun* precuneus, *INS* insula, *FFG* fusiform gyrus, *PSCG* postcentral gyrus, *PMC* premotor cortex, *PCC* posterior cingulate cortex, *DLPFC* dorsolateral prefrontal cortex, *MTG* middle temporal gyrus, *IPL* inferior parietal lobule, *Thal* thalamus, *IOG* inferior occipital gyrus

Among exteroceptive-processing regions, PCun seems to play an important role. In particular, beyond the previous described reduced activity in all interoceptive domains, i.e., heart, stomach and bladder sensations, PCun shows diminished activations during olfactory, tactile and gustatory perception [68, 70, 71]. It only shows increased activity during heat perception [65]. Finally, mental-self-processing regions are reported to have differential activity: i) enhanced activity in left MTG and right DLPFC during gustatory stimulation [66, 67], and in PCC when experiencing thermal stimuli [65, 72] and ii) decreased activity in left DLPFC and PCC during tactile stimulation [71] (Fig. 5, upper part).

### Body image tasks—abnormal responsivity to own’s and others’ body images

Body image distortion is a hallmark feature of AN. The subjective perception of body weight or shape is disturbed, together with increased attention to particular details or parts of the body [2]. Body image disturbances are negatively correlated with patients’ long-term outcomes of remission [76]. Various fMRI studies had investigated neural responses in AN when viewing body images using four different paradigms: (i) self-body images; (ii) thinner self-body images; (iii) fatter self-body images; and iv) other-body images.

Neural activity displays a different activity in response to the four different stimuli. When images of their own body are shown to patients, no significant activation was found, whereas control subjects showed an activation of posterior regions such as occipital cortex, PCC, PCun and some temporal regions [77, 78]. Furthermore, the same studies observed a decrease in neural activity in left IPL and PCun [77, 78]. On the contrary, Via and colleagues (2018) found increased activity in dorsal PCC respect to controls when patients were looking at photographs of their own bodies [55]. Moreover, when patients looked at distorted images of their own bodies (thinner and fatter) and when looking at other women’s bodies, their neural activity is found to be generally increased [79–82].

The fMRI studies in this field have highlighted only enhanced activations in (i) bilateral insula, bilateral IPL, PSCG and bilateral thalamus among interoceptive-processing regions [77, 78, 80, 83, 84], (ii) MPFC, PCun, right FFG and left IOG among exteroceptive-processing regions [79–81], and (iii) bilateral DLPFC, bilateral MTG and PCC among mental-self-processing regions [77, 78, 82, 83]. Only one study, in patients with AN (in comparison with the HC group), showed less activation of the rostral ACC induced by the self-other body-shape comparison [83] (Fig. 5, lower part).

### Summary of task-related activity in the three layers topography of self regions

Together, the studies concerning the task-related activity show how (i) interoceptive-processing regions have an overall increased activity across both intero-exteroceptive and body image tasks; (ii) the three types of self-processing regions are hyperactivated when body images are shown, whether they are of one’s own body (especially if distorted) or of other subjects’ bodies. This strongly suggests decreased sensitivity of all three layers of self to the distinction of self- and non-self-specificity related to the body.

### Rest-task study

We observed an overall decreased functional connectivity at rest between the three levels of self-processing regions and an overall increased activity in the same regions during the different task-related activities, especially in the regions responsible for the interoceptive processing and the interoceptive self (insula, thalamus, PSCG, ACC and IPL). In order to understand if there is a relationship between resting hypoconnectivity and hyperactivity during the task, we therefore analyzed the studies that apply the same paradigms on the same sample of patients.

Among the fMRI studies in the literature, only one applied a rest-task design on the same AN and healthy subjects by applying a task among those previously considered. Via and colleagues (2018) [55], using a seed-based approach, found a decreased intra-DMN rsFC between PCC and left AG. Subsequently, once a body image task was applied in which images of their own bodies and bodies of other subjects were shown to the AN patients, they observed how patients with AN showed increased activity in the dorsal PCC respect to controls, when looking at their own bodies, as well as a failure to activate the PCun and ventral PCC in response to processing another’s body. Moreover, a pattern of increased functional task-related connectivity with the DACC for the self and with mid-temporal areas for the other condition was also observed [55].

This study supports the co-occurrence of decreased rsFC (from midline to lateral cortical regions) with increased task-related activity in the same regions. This co-occurrence is also compatible with the findings in the separate rest and task studies described above showing decreased SCMS rsFC and mostly hyperactivation during task states.

## Discussion

What is the source of the body-self disruption reported in AN patients? To address this yet open question, we conducted systematic fMRI review of resting state, task-evoked, and rest-task studies in AN. The extrapolated data summarized in the present review are the following: (i) decreased rsFC of SCMS in AN patients compared to controls; (ii) reduced rsFC between medial (DMN and SN) and lateral (ECN) cortical regions in AN; (iii) decreased rsFC in mainly the regions of the interoceptive self in AN; (iv) altered activity with overall increased activity in response to sensory and body image stimuli in patients, especially in the regions of the interoceptive self; and (v) lack of a clear task-related distinction between own’s and others’ body image in AN.

Brain imaging studies indicate that self-referential processing (SRP) is elaborated largely in the SCMS and other limbic cortices, such as the insula and ACC [85–88]. During SRP, self-related information is propagated beginning from the internal body to the external environment and is thus integrated within the self [36]. This highlights the importance of the interoceptive layer as a cornerstone from which self-processing begins and allows adequate integration of self-related information into the self. This type of information is related to subcortical and limbic structures such as thalamus, insula and ACC, which receive afferents directly from the whole body [11, 36], and subsequently communicate with the midline cortical regions in order to integrate them into a sense of self [88–90].

Despite using different methods when analyzing different variants of rsFC, for example, independent component analysis (ICA) and graph theory, all approaches demonstrate similar findings, namely decreased rsFC among SCMS and altered rsFC in insula and ACC. These data might suggest that an alteration in the interoceptive-self layer may be an important factor in AN etiopathogenesis. More in detail, subcortical structures (e.g., thalamus, caudate, putamen) show a lack of synchronization (i.e., reduced rsFC) with various cortical midline regions (e.g., Ant.PFC, VMPFC, MOFC, PCC, PCun) and a reduced rsFC between thalamus and insula has been systematically observed [39–41], highlighting a lack of subcortical–cortical communication within interoceptive-self regions already present at rest in patients with AN. These data show reduced rsFC in the interoceptive-processing regions and confirm a reduced rsFC between regions belonging to DMN, SN and ECN, as widely discussed in literature [51, 56, 70]. The fact that several studies have identified a diminished interoceptive ability, both across interoceptive accuracy and awareness, in patients with AN [9, 17–19], further underlines the key role of an altered interoception of the own body in AN.

In addition to reduced subcortical–cortical interoceptive resting state activity, examined data highlights an overall increased task-related activity in response to AN-specific tasks about multisensory integration and body image. Such task-related hyperactivity occurs in the same regions showing reduced activity and functional connectivity at rest. This may suggest that decreased resting state activity in these regions prone them to react abnormally strong to external stimuli or tasks [91, 92]. This brain dynamics does not seem to be a peculiarity of the AN condition: negative rest-task modulation has also been found in patients with social anxiety disorder (SAD) [93]. Interestingly, AN and SAD are disorders that often occur in comorbidities (55%), with the anxiety disorder tending to be already present prior to the onset of the eating disorder [94]. The percentage increases if we also consider other anxiety disorders, e.g., generalized anxiety disorder (GAD), panic disorder (PD): 83% of patients with AN have reported at least one lifetime diagnosis for an anxiety disorder [94]. This emphasizes the importance of anxiety in the etiopathogenetic mechanism of AN, with neural consequences that might be similar to those reported in anxiety disorders.

**Fig. 6:**
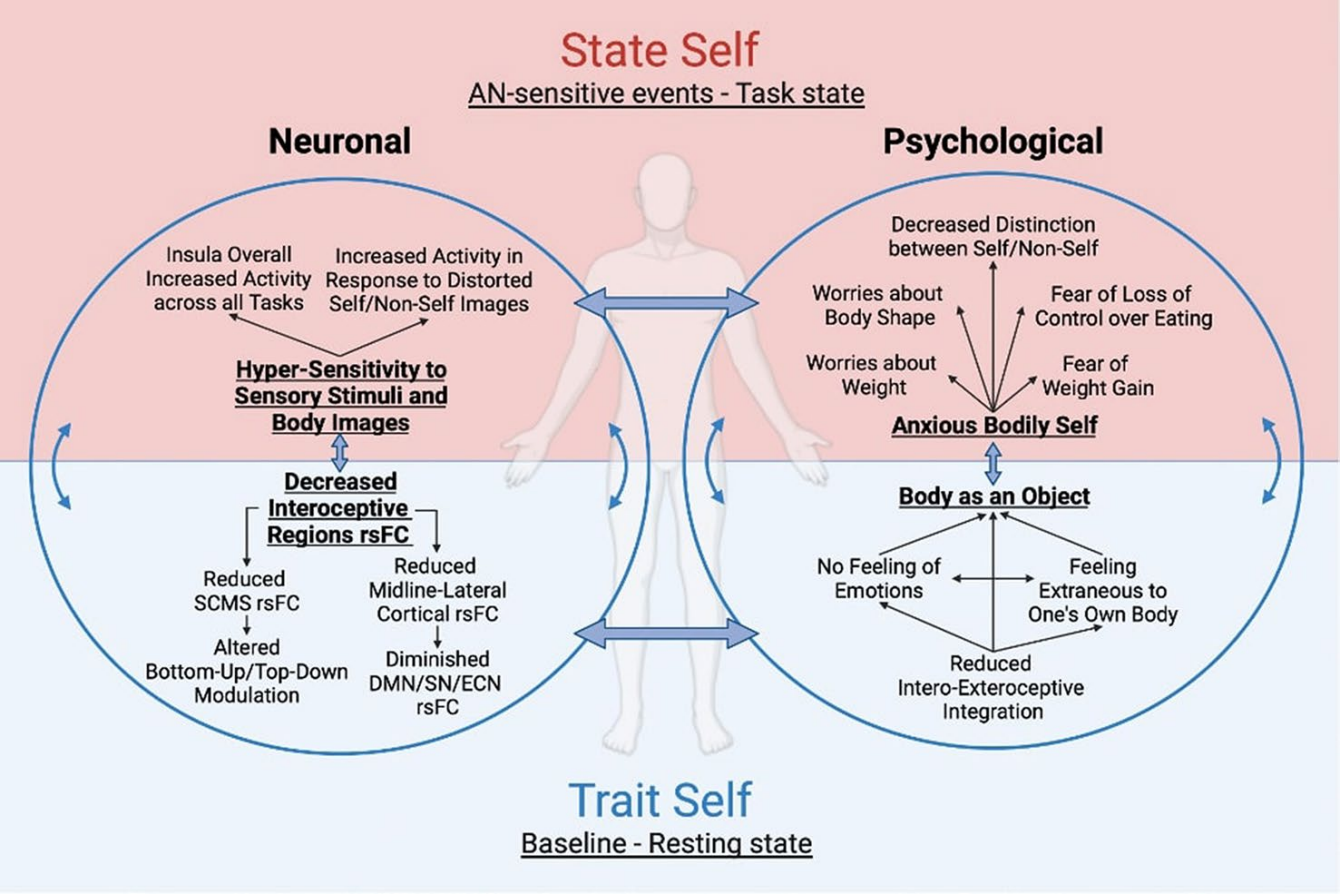
Model of neuronal (left circle) and psychological (right circle) features of patients with AN divided in resting state activity, namely “trait self” (lower), and task-related activity, namely “state self” (upper)

Together, the analyzed data also show overall increase in task states in patients with AN when exposed to images of their own and others’ body across the three-layers-of-self regions. Intriguingly, these patients seem not to respond to images of their own not distorted body, i.e., as it appears, showing no activity at all or decreased activity (in IPL and PCun) [77, 78]. This lack of activity becomes more interesting if, alternatively, when distorted images of one’s own body (fatter and thinner) and of the body of others are shown, hyperactivity of the three-layers-of-self regions is reported, underscoring a high sensitivity toward body images not related to the real appearance of one’s body which may be related to body dysmorphic distortions in patients with AN [31]. This anxiety related to bodily stimuli may reflect excessive attention to the body by these patients, highlighting once again the key role of the bodily/interoceptive self in AN (Fig. 6).

How do these neural alterations shape the phenomenal features displayed by these patients resulting in psychopathological symptoms? The main symptoms of AN revolve around the body: a strong fear of gaining weight, excessive worries about body shape and weight, and a distorted view of one’s body [1]. In addition, these patients report an altered emotional experience, characterized by a difficulty in recognizing and expressing emotions, namely alexithymia or unstable emotions [6, 95, 96]. Given the interdependent nature of physiological and subjective emotional states, the extent to which an individual can detect and interpret bodily signals has been considered influential in shaping emotional experiences [97–99]. This is consistent with theories that suggest that physiological responses in the body are the origin of emotions, i.e., embodiment of emotional feelings [100, 101]. Precisely, the foundation for our emotional feelings lies in the neural representation of the physiological condition of the body, with “somatic markers” evoking feeling states that influence cognition and behavior [100, 102]. Following these theories and given the reported reduction in interoception, AN subjects may not perceive the physiological correlates of the interoceptive sensations, and thus they may not experience, identify and express related emotions. Clinical practice also seems to confirm this suggestion. In fact, AN patients appear to be emotionally cold, as if the affective component was flattened and detached from the sense of self [103, 104], which alternatively becomes defined by the other’s gaze and external appearance [66]. They feel weakly anchored in their body and alienated from it and from their own emotions [104], which may result in an inability to perceive bodily stimuli as self-specific engendering an objectification of the body, i.e., body as an object [5, 31, 33]. Hence, not being able to integrate emotions into one’s self might be the mechanism that underlies the body-self disruption typical of AN, namely altered emotional self-consciousness.

Finally, patients with AN are reported to have a strong anxious activation for those stimuli related to hunger and food that may mirror an attempt to control body shape and weight as the most important goal [1]. Accordingly, the extrapolated data highlight an overall increased whole-brain activity in response to AN-specific stimuli, such as interoceptive and exteroceptive stimuli and body images, especially in the interoceptive-self regions with reduced rsFC. The reported hyperactivity in AN patients concerns a reaction to stimuli related to the body, i.e., bodily self, which are actually related to fear and anxiety activations underlining AN core symptoms, such as feeling hungry, perception of tastes and the fear of gain weight [1]. Thus, an anxiety-inducing stimulus may determine an increased activation in the same abnormal resting state regions, resulting in an enhanced attention for one’s own body, i.e., anxious bodily self [105]. Even more so if at rest there is already an altered bottom-up/top-down modulation between the subcortical medial regions (involved in interoception and emotional experience) and the cortical medial regions (involved in SRP and cognitive processing), as happens similarly in anxiety disorders (Fig. 6) [93, 106].

Given the ineffectiveness of pharmacological treatment in patients with AN [107], these observations could have important clinical implications for psychotherapeutic treatment. While cognitive-behavioral therapy (CBT) strategies such as exposure to hierarchies of “forbidden” foods, supervised meal exposures and body shape/mirror exposure are already extensively studied in AN-specific CBT [108], recent AN treatment studies highlighted the prominent role of targeting bodily experiences and emotional dysregulation, such as fear and anxiety [109, 110], in determining an improvement in specific eating psychopathology. Future studies are required to investigate whether a body-centered therapy, i.e., interoceptive exposure (IE), could lead to an improvement in AN symptomatology and its pre-post-treatment neural correlates.

### Limitations

The present systematic review has limitations that need to be considered. Firstly, it is important to note that the diversity in the analysis and methodologies (such as seed-based approach, ICA, graph theory in resting state) within the studies included in this systematic review precluded the completion of a meta-analysis. Furthermore, the studies we reviewed reported differences in the analyzed samples in terms of diagnosis of AN. In fact, respectively to the 46 studies reviewed in the present systematic review, the total sample of patients with AN analyzed amounts to 893 subjects. Of these, 638 patients (71.4%) reported a diagnosis of restrictive subtype AN in 37 studies, 85 patients (9.5%) shown binge-purging subtype AN in 15 studies, whereas 170 patients (19.1%) were not given a specific diagnosis of a particular subtype of AN in nine studies. Representing 71.4% of the total sample, there is a disproportion toward AN patients with the restrictive subtype versus the binge-purging subtype that needs to be taken into account. Secondly, the examined studies report different illness durations. This variable comprehensibly plays an important role in the brain of patients with AN and suggests caution in drawing inferences. Thirdly, another limitation is represented by the presence of confounding factors in AN neuroimaging such as nutritional status (malnourishment), brain development and hydration that must be taken into consideration. Indeed, these factors might affect the neuronal data with consequences in their interpretation (King et al., 2018). Nevertheless, the differentiation between alterations subsequent to the disease/malnourishment and those causally involved in the etiopathogenesis of the disorder is still a big challenge for the neuroscientific research. Finally, in some of the analyzed studies, patients reported different comorbidities and drug treatments that needs to be considered as other potential confounds.

## Conclusion

The synthesis of the evidences collected in this paper seems to converge in the hypothesis that AN might be considered as a basic disturbance of the interoceptive self-generated by an alteration of the neural interoceptive-processing regions. The well-known symptoms of AN could therefore be due to neural alterations already present in the resting state that influence brain activity when stimulated by a task. Interoceptive (body) deficits and lack of communication with cortical areas may predispose AN patients to greater top-down cognitive (mental) rigidity, in order to control their body. This body-mind dissociation in patients with AN may lead to high levels of distrust toward their own body and its sensations, i.e., body mistrust. This underlines the importance of a therapy that targets the body, its sensations and its integration into a sense of self in AN.

Reduced interoception might have a role in creating negative affective states which contributes to top-down modulatory influences and subsequent focus on long-term goals rather than short term needs. This is compounded by well-established deficits in reward processing, leading to a reduced reliance on intuitive responses and thereby the perpetuation of negative eating behaviors and intentional denial of illness. These observations may have important clinical implications. A body-centered therapy, i.e., IE, could help patients with AN to recover an adequate emotional experience that can be integrated in the sense of self with psychological and physical benefits. Hence, typical CBT strategies might include a more explicit integration of IE in order to obtain an enhanced bodily self-consciousness in patients with consequences on AN symptomatology.

### What is already known on this subject?

In recent years, there has been a growing body of evidence associating eating disorders to altered interoception, underlining the importance that is attributed to the body in these patients. Several studies have identified an altered interoception in its objective and subjective measurements in patients with AN, hypothesizing that deficits at the interoceptive level may play a role in typical symptoms of eating psychopathology such as restriction, emotional dysregulation and body image disturbances. Nevertheless, very few studies have analyzed neuronal activity through interoceptive paradigms using fMRI. In addition, no previous systematic review has synthesized the available evidence on the neuronal link between eating psychopathology and interoception across rest and task states.

### What does this study add?

To try to shed light on how neuronal activity in patients with AN may be associated with eating psychopathology, we examined neural activity investigated by studies during resting and task states. We considered resting state functional connectivity and the resulting neuronal activity activated by AN-specific tasks with a focus on regions involved in interoception. The division of brain regions tracked the topography of the three-layer model of self-processing. The data showed an alteration of the neural interoceptive-processing regions, particularly between subcortical and anterior midline cortical regions (SCMS), suggesting how interoceptive deficits may play a role in altered emotional experience and body-objectification. These observations may have clinical implications, prompting the use and development of body-centered/interoceptive psychotherapeutic techniques to help patients with AN more effectively.
